# Draft Genome Sequence of *Clostridium beijerinckii* Ne1, Clostridia from an Enrichment Culture Obtained from Australian Subterranean Termite, *Nasutitermes exitiosus*

**DOI:** 10.1128/genomeA.00303-15

**Published:** 2015-04-23

**Authors:** Han Wang, Hai Lin, Nai Tran-Dinh, Dongmei Li, Paul Greenfield, David J. Midgley

**Affiliations:** a University of Science and Technology, Beijing, China; b CSIRO, Food & Nutrition Flagship, Dickson, Australia; c Plant Health & Environment Laboratory, Ministry for Primary Industries, Auckland, New Zealand; d CSIRO, Digital Productivity and Services, Dickson, Australia; e CSIRO, Energy Flagship, Dickson, Australia

## Abstract

The draft genome of *Clostridium beijerinckii* strain Ne1 was reconstructed from the metagenomic sequence of a mixed-microbial consortium that produced commercially significant quantities of hydrogen from xylan as a sole feedstock. The organism possesses relatively limited hemicellulolytic capacity and likely requires the action of other organisms to completely degrade xylan.

## GENOME ANNOUNCEMENT

The termite hindgut has been described as the world’s smallest bioreactor (1). Within the gut, lignocellulose is digested by the host in conjunction with its microbiome, and various byproducts, including hydrogen, are produced in the process (2). The production of biogenic hydrogen represents a promising alternative energy source, as it is environmentally sound, inexpensive, and possesses a high energy density (3). One recently derived hydrogenogenic mixed-microbial culture (designated 1 TC) obtained from the gut of a worker *Nasutitermes exitiosus* (collected 33°45′34″S; 150°59′25″E), was capable of producing commercially significant quantities of hydrogen, with little CO_2_, using xylan as a feedstock. The 1 TC metagenome was sequenced using Illumina HiSeq 2000 and assembled using Velvet 1/1/07 (4). The 1 TC culture was composed, almost exclusively, of three clostridial taxa which were named Ne1, Ne2, and Ne3 after *N. exitiosus*. Contigs belonging to these three genomes were separated (5) and checked manually. Ne1 was a strain of *Clostridium beijerinckii*, Ne2 was a novel taxon related to *Clostridium magnum* (6), while Ne3 was a *Ruminiclostridium* species (7). This paper describes the genome of Ne1.

Ne1 was the second most abundant clostridia in the 1 TC consortia, accounting for 27% of the reads in the 1 TC metagenome. In total, the draft Ne1 genome includes 290 large (>200 bp) contigs which total ~5.6 Mbp in length, a size similar to previously published *C. beijerinckii* genomes (8–10). The longest contig was ~110 kbp, and the size distribution of the other contigs had mean, median, and *N*_50_ lengths of 19,163 bp, 12,981 bp, and 25,991 bp, respectively. Annotation was performed using Integrated Microbial Genomes Expert Review (IMG-ER) (11), which predicted a total of 5,004 protein-coding genes and 27 structural RNAs. The annotated genome is available for download at IMG-ER (http://img.jgi.doe.gov/mer), and the sequences and metadata are available at the European Nucleotide Archive under accession no. PRJEB8629 (http://www.ebi.ac.uk/ena/data/view/PRJEB8629).

We speculate that Ne1 likely plays a role in dark fermentation of pentose sugars to hydrogen in the consortia but is not the primary degrader of xylan. Analysis of the Ne1 using dbCAN (http://csbl.bmb.uga.edu/dbCAN/index.php) (12) reveals that the genome does not appear to encode many enzymes involved in the degradation of xylan. Complete digestion of xylan requires endo-β-1,4-xylanase, β-xylosidase, and several accessory enzymes, including α-l-arabinofuranosidase, α-glucuronidase, acetylxylan esterase, ferulic acid esterase, and a p-coumaric acid esterase (13). Ne1 appears to have genes mostly involved in cleaving side branches (GH51, GH67) from the xylan molecule and for oligosaccharide catabolism (e.g., GH1, -2, -3), it appears to lack specific xylanases. It is noteworthy that Ne1 does possess a gene that encodes an enzyme from GH43; however, the majority of enzymes in this class have activities against oligosaccharides rather than intact xylan. Work to understand the roles of Ne1, Ne2, and Ne3 in producing biohydrogen from xylan is ongoing.

### Nucleotide sequence accession numbers. 

This whole-genome shotgun project has been deposited at DDBJ/EMBL/GenBank under the accession numbers CEMD01000001 through CEMD01000290.
